# Supplementary material for: Petroleum exploration increases methane emissions from northern peatlands
Source: Nat Commun. 2019 Jun 26;10:2804. doi: 10.1038/s41467-019-10762-4 (PMC6594948; doi:10.1038/s41467-019-10762-4)
Supplement: Supplementary file 1 — Supplementary Information [file 41467_2019_10762_MOESM1_ESM.pdf]

Supplementary Information

Petroleum exploration increases methane emissions from northern peatlands

Strack *et al*.

Supplementary Table 1: Comparison of peatland seismic line length between ABMI Human Footprint database and ABMI Enhanced Linear Features database[a]

	Bog	Fen	Swamp	Peatland Total
**Total area (km²)**	711	3339	837	4887
**Seismic line length from ABMI Human Footprint database[1] (km)**				
**Legacy + trails[b]**	1418	5693	1097	8207
**LIS[b]**	441	1651	377	2469
**Total**	1859	7344	1474	10676
**Seismic line length from ABMI Enhanced Linear Feature database[2] (km)**				
**Legacy + trails[b]**	2011	8190	1546	11747
**LIS[b]**	1320	5770	1137	8227
**Total**	3331	13960	2683	19974
**Seismic lines included in ABMI Human Footprint database (%)**				
**Legacy + trails[b]**	70	70	71	70
**LIS[b]**	33	29	33	30
**Total**	56	53	55	53

a. Numerical results from our comparison of the ABMI Human Footprint database[1] used throughout the study to a higher resolution data set under development at ABMI (ABMI Enhanced Linear Features)[2], using mapped seismic line data with 1 m spatial resolution air photos in a site located near the town of Conklin, Alberta, Canada (55.6314° N, 111.0839° W). Total study area for comparison is 10,475 km² containing 4,887 km² peatland.
b. LIS = low-impact seismic lines. Description of legacy lines and trails is given in Methods

Supplementary Table 2: Length of seismic lines within wetland types for the province of Alberta

Wetland Type	Bog	Fen	Marsh	Swamp	Open Water	Total
**Total Area Covered (km²)**	30048	58580	16039	46155	20073	170895
**LIS (km)**	9093	17449	144	7515	22	34223
**Legacy lines (km)**	73437	112627	8088	92016	1408	287576
**Trails (km)**	6049	14508	2580	12950	696	36782

Supplementary Table 3: Area of different types of seismic Lines within wetland types for the province of Alberta

Wetland Type	Bog	Fen	Marsh	Swamp	Open Water	Total
**LIS (km²)**	27.3	52.3	0.4	22.5	0.1	102.6
**Legacy (km²)**	440.6	675.8	48.5	552.1	8.4	1725.4
**Trails (km²)**	24.2	58.0	10.3	51.8	2.8	147.1

Supplementary References

1. Alberta Biodiversity Monitoring Institute (ABMI), *Wall-to-Wall Human Footprint Inventory 2014*. (Alberta Biodiversity Monitoring Institute and Alberta Human Footprint Monitoring Program, Edmonton, AB, Canada, 2017).
2. Alberta Biodiversity Monitoring Institute (AMBI). *Enhanced Linear Features in the Oil Sands Region 2014* (Alberta Biodiversity Monitoring Institute, Edmonton, AB, Canada, 2017).
