# Supplementary material for: Petroleum exploration increases methane emissions from northern peatlands
Source: Nat Commun. 2019 Jun 26;10:2804. doi: 10.1038/s41467-019-10762-4 (PMC6594948; doi:10.1038/s41467-019-10762-4)
Supplement: Supplementary file 3 — Description of Additional Supplementary Files [file 41467_2019_10762_MOESM3_ESM.pdf]

Description of Additional Supplementary Files

File Name: Supplementary Data 1
Description: Compiled peatland methane fluxes for western Canada for bogs and fens and Canada for swamps (n.r. = not reported for latitude and longitude). Reference numbers refer to those in the main manuscript. See accompanying .txt file.
